# Novel Exons and Splice Variants in the Human Antibody Heavy Chain Identified by Single Cell and Single Molecule Sequencing

**DOI:** 10.1371/journal.pone.0117050

**Published:** 2015-01-22

**Authors:** Christopher Vollmers, Lolita Penland, Jad N. Kanbar, Stephen R. Quake

**Affiliations:** 1 Dept. of Bioengineering, Stanford University, Stanford, California, United States of America; 2 Dept. of Applied Physics, Stanford University, Stanford, California, United States of America; 3 Howard Hughes Medical Institute, Chevy Chase, Maryland, United States of America; Chang Gung University, TAIWAN

## Abstract

Antibody heavy chains contain a variable and a constant region. The constant region of the antibody heavy chain is encoded by multiple groups of exons which define the isotype and therefore many functional characteristics of the antibody. We performed both single B cell RNAseq and long read single molecule sequencing of antibody heavy chain transcripts and were able to identify novel exons for IGHA1 and IGHA2 as well as novel isoforms for IGHM antibody heavy chain.

## Introduction

Sequencing and annotation of the human immunoglobulin constant regions was largely accomplished in the 1980s and early 1990s. The IGH locus was mapped to the telomeric region of the q arm of chromosome 14 [1,2,3], the sequence of the different isotype exons in the constant region was determined [4,5,6,7], and alternative splicing was confirmed as the mechanism for the expression of both membrane bound and secreted isoforms of all isotypes [8,9]. After these accomplishments attention switched to the variable region [10,11]. More recently high throughput sequencing has greatly increased our knowledge of diversity in the variable region by the in-depth sequencing of antibody repertoires [12,13,14]. Three decades of intense scrutiny, in conjunction with the completion of the human genome project [15,16], led to the assumption that the genome sequence and transcriptional products of the human immunoglobulin locus were completely characterized.

Each isotype (IGHM,IGHD, IGHG1-4,IGHA1-2,IGHE) was thought to be expressed as one of two isoforms: secreted or membrane bound which contain S or M exons respectively [8,9,19]. Transcripts of all isotypes are thought to feature two exons specific to their membrane isoform, with the exception of IgA1 and IgA2 (transcribed from IGHA1 and IGHA2) which were believed to feature only one membrane exon[20].

Analysis of the IGH locus and its transcripts is highly challenging due to the fact that every IGH transcript is the result of a unique somatic recombination event and can be expressed as different isotypes and isoforms. While RNAseq has been proven to be a very powerful tool for the discovery and annotation of transcript isoforms, standard RNAseq protocols rely on RNA pooled from thousands to millions of cells. As large pools of B cells will contain a highly diverse mixture of IGH transcripts standard RNAseq would result in an even more diverse pool of short read fragments that would be impossible to de-convolute. We used two strategies to analyse IGH transcripts in more detail than previously possible. First, we used single B cell RNAseq [17,18] to reduce the complexity down to a single IGH transcript. Second, we used single molecule long read sequencing to analyze single IGH transcripts amplified from a bulk B cell RNA sample.

Using these strategies, we discovered novel exons in the 3’UTR of IGHA1 and IGHA2 transcripts. We also found that in addition to the canonical secreted and membrane isoforms, all IGH isotypes and especially IGHM showed further, less abundant shorter isoforms.

## Results

### Novel non-coding exon in IGHA 3’UTR

**Figure 1:**
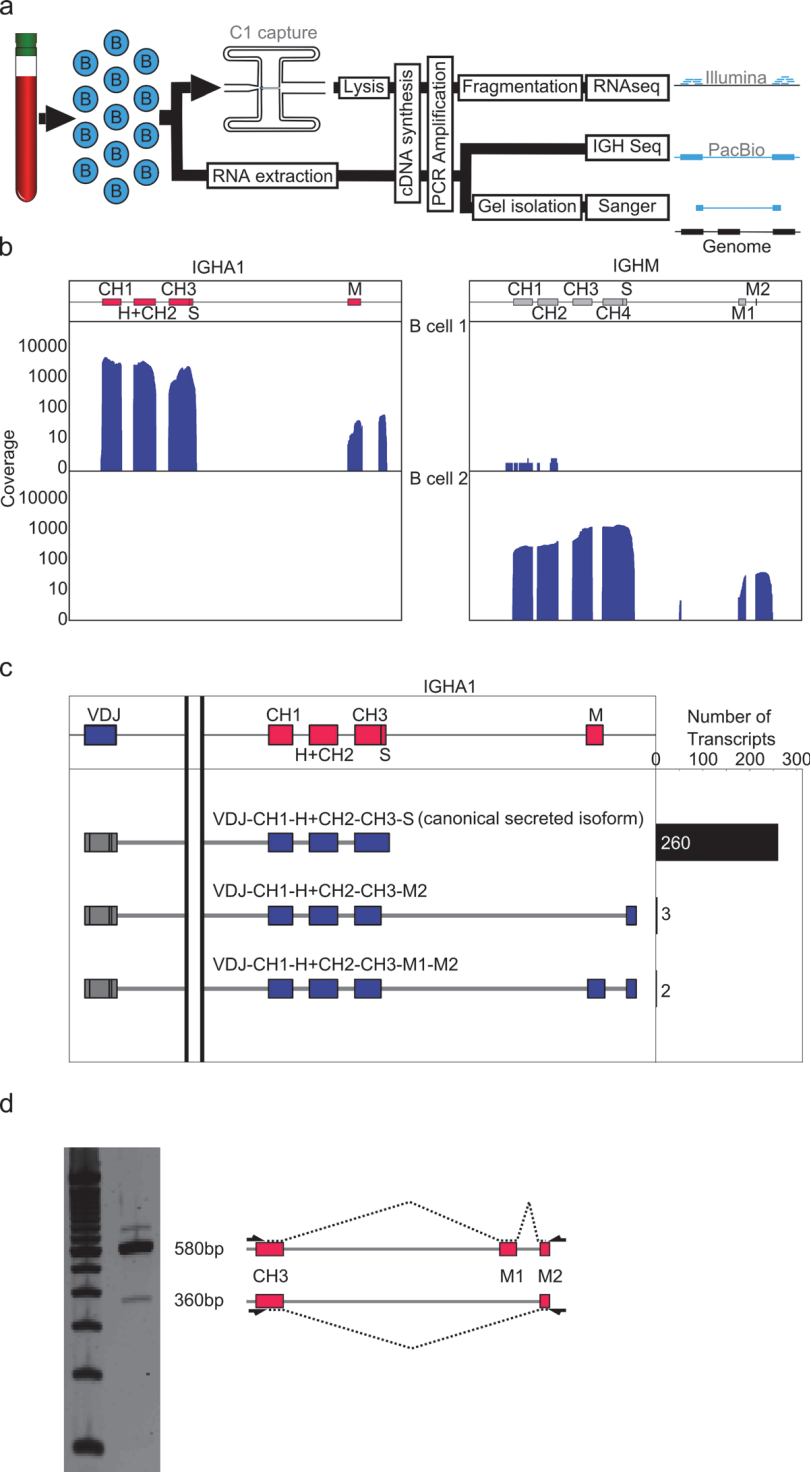
IGHA 3′ UTR contains novel splice junction. a) Full length cDNA of single B cells was generated using the C1 autoprep system, fragmented using Nextera XT, and sequenced using Illumina sequencers (RNAseq). Additionally, RNA was extracted from bulk B cells and the IGH transcripts were analyzed using the PacBio sequencer (IGH-Seq) or conventional Sanger sequencing after gel isolation b) RNAseq reads of two B cells were aligned to the IGH locus. Coverage density is shown as a histogram for both IGHA1 and IGHM exons for both B cells. Coverage density in the IGHA1 expressing cell indicated a splicing event in the canonical IGHA membrane exon. c) PacBio single molecule sequencing reads were mapped to the IGHA1 locus. Reads containing the whole VDJ region as well as either S or M exons were grouped and quantified. This confirmed the presence of a splice site in the canonical IGHA1 membrane exon resulting in two exons (named IGHA1 M1 and M2). d) Gel separation of amplicons generated from bulk B cell RNA using primers specific for exon J4 and putative exon IGHA1 M2 on the left. Schematic representation of isoform splice structure on the right. The longer band confirmed the IGHA1 M1 to M2 splicing event, the shorter band represents a novel isoform of IGHA1.

We applied single cell RNAseq to human B cells to analyse complete variable and constant regions of IGH transcripts expressed by single B cells (Fig. 1A).

For single B cell RNAseq, we amplified full length cDNA from single B cells using the C1 Autoprep system and used Nextera XT transposase to generate libraries which we sequenced on the Illumina MiSeq and HiSeq Sequencers. When we mapped the resulting RNAseq reads to the IGH locus we identified a B cell expressing high levels of both secreted and membrane bound isoforms of IGHA1. Interestingly, instead of one continuous exon as previously assumed (IGHA1 M), this cell appeared to have two exons (subsequently named IGHA1 M1 and IGHA1 M2) in the membrane isoform, with the second located in the 3′UTR. While the non-coding transcript AL928768.3 containing only IGHA1 M1 and IGHA1 M2 has previously been identified, the expression of both IGHA1 M1 and IGHA1 M2 appeared to be dependent on IGHA1 expression as the exons were absent in a B cells expressing IGHM exclusively (Fig. 1B). Assembly of the RNAseq reads confirmed high confidence splice sites between IGHA1 M1 and M2 as identified by SplicePort [21] (S1A Fig.). This showed that the first 236bp exon (IGHA1 M1, chr14:106170704-106170939) codes for the complete transmembrane region and is spliced, excising a 338bp intron, to a second 122bp non coding exon (IGHA1 M2, chr14:106170301-106170423).

While single B cell RNAseq provided a strong indication about the isoform structure of membrane bound IGHA1 we aimed to validate this result using single molecule long read sequencing[17,18] (Fig. 1A). In contrast to RNAseq single molecule long read sequencing provides continuous sequencing reads covering complete IGH transcripts, enabling us to validate splice junctions with high confidence. We amplified cDNA from bulk B cell samples by priming of the V leader segments as well as the polyA tail of IGH transcripts. We sequenced the resulting amplicons on a PacBio RS II sequencer generating 1077 full length IGH transcripts containing either S or M exons. We identified 265 transcripts featuring isoforms of the IGHA1 isotype. Most IGHA1 transcripts were of the canonical secreted isoform (260), but two membrane isoform transcripts validated the existence of IGHA1 M1 and IGHA1 M2 (Fig. 1B, S1A Fig.). We also observed the corresponding exon structure and isoforms in IGHA2 (IGHA2 M1, chr14:106050470-106050705 and IGHA2 M2, chr14:106050066-106050190) (S1C Fig.).

Finally, we also performed PCR on cDNA from an independent individual using primers in the IGHA1 CH3 exon as well as the IGHA1 M2 exons and detected a band of the expected size for the VDJ-CH1-CH2-CH3-M1-M2 isoform (Fig. 1C).

Taken together, we used RNAseq, single molecule read sequencing and cDNA size separation to conclusively show that membrane bound IGHA1 and IGHA2 transcripts undergo a previously unknown splice event. This splicing event creates a non-coding exon in their 3’UTR.

### Novel isoforms of IGHM transcripts

Surprisingly, when we gel separated the IGHA1 constant region cDNA, we also detected a shorter band (Fig 1D) and Sanger sequencing confirmed that it represented an isoform in which the CH3 exon is spliced directly to the IGHA1 M2 exon (VDJ-CH1-CH2-CH3-M2) (S2B Fig.). Indeed, three transcripts in our single molecule long read sequencing data corresponded to this previously unknown isoform (Fig. 1C). By skipping the stop codon in the IGHA1 M1 exon this isoform creates a novel reading frame terminating in a stop codon shortly before the polyA signal.

**Figure 2:**
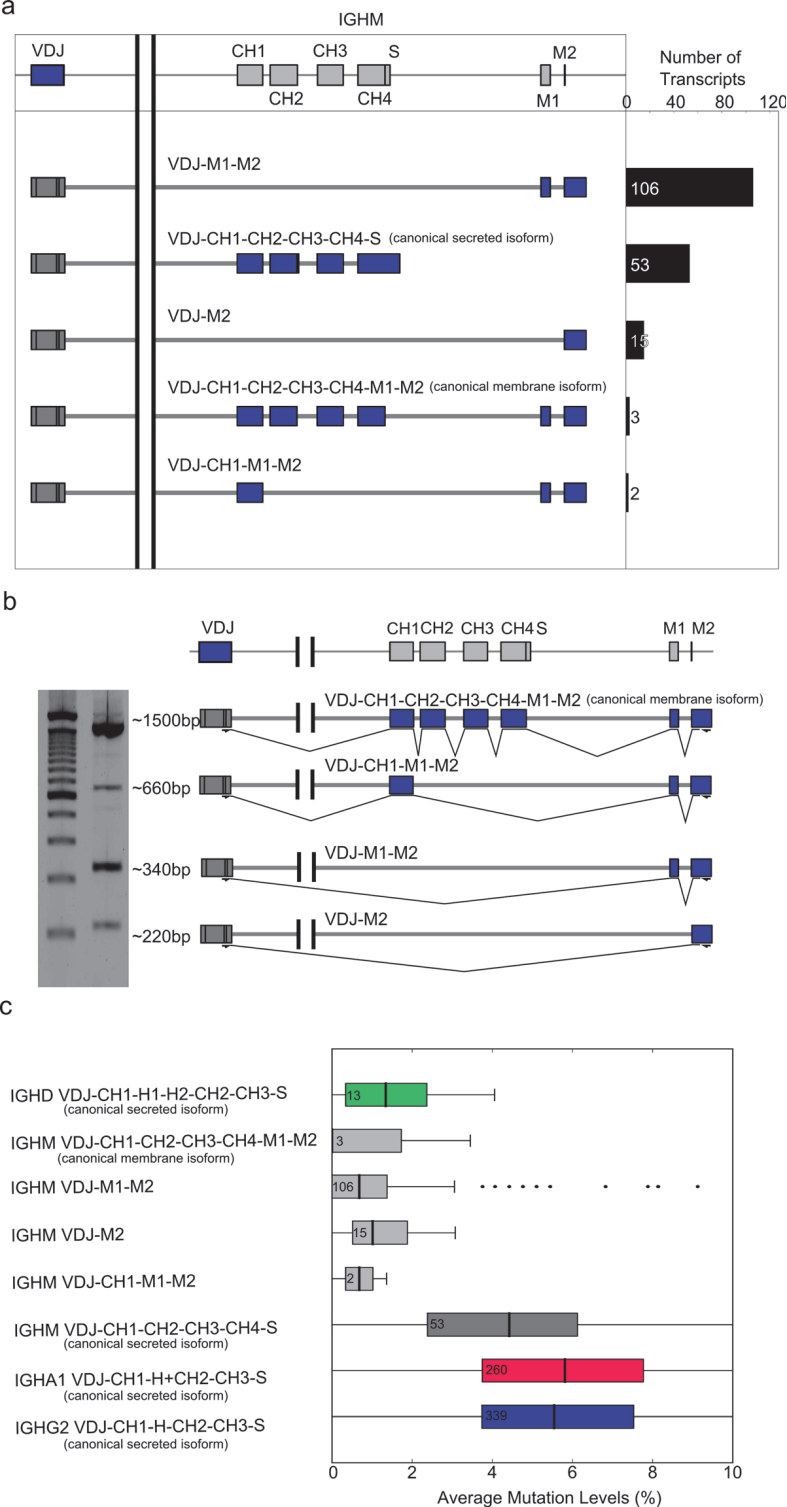
Alternative splicing of IGHM transcripts. a) RNA was extracted from bulk B cells and the IGH transcripts were analyzed using the PacBio sequencer (IGH-Seq) and mapped to the IGHM locus. Reads containing the whole VDJ region as well as either S or M exons were grouped and quantified. Several reads represented abundant novel short IGHM isoforms lacking structural exons. b) Gel separation of amplicons generated from bulk B cell RNA using primers specific for exon J4 and putative exon IGHA M2 is shown on theleft. Schematic representation of isoform splice structure as validated by Sanger sequencing is shown on the right. The bands of several sizes confirmed the presence of short IGHM isoforms found by single molecule sequencing. c) Mutation rates of different IGH isoforms derived from PacBio reads shown as boxplots. The low mutation rates of the novel isotypes strongly indicate that their expression is limited to naïve B cells.

As the single molecule long read sequencing data was not limited to IGHA transcripts, we expanded our analysis to IGHM. Indeed, IGHM transcripts showed abundant expression of previously unknown isoforms composed of known exons. In addition to 56 transcripts featuring canonical isoforms, we identified three previously unknown isoforms of the IGHM transcript (Fig. 2A), which were also validated using PCR and Sanger sequencing (Fig. 2B). The first and at 106 transcripts most abundant new isoform (VDJ-M1-M2) contained only the M1 and M2 exons spliced directly to the VDJ exon (Fig. 2A). The sequence of this isoform as derived from the consensus of single molecule reads and validated through Sanger sequencing confirmed an in-frame splicing event that leads to a functional transmembrane domain (S2 Fig.). The second most abundant novel isoform (VDJ-M2) contained only the M2 exon spliced directly to the VDJ exon. In this case the splice junction is out-of-frame, generating a novel reading frame (S2 Fig.). The third and least abundant isoform (VDJ-CH1-M1-M2) contained CH1, M1, and M2 exons and once again encoded a functional transmembrane domain (S2 Fig.).

To identify a target B cell population expressing the more abundant, novel IGHM isoforms we quantified the rate of hypermutation in the V region of these transcripts. Different population of B cells express distinctly different IGH transcripts. Naïve B cells express predominantly membrane bound IGH transcripts that are non-mutated and of either IGHM or IGHD isotype. Activated B cells express predominantly secreted IGH transcripts that is mutated and of either IGHM, IGHA(1–2), IGHG(1–4), or IGHE isotype.

The 3 novel isoforms we observed (VDJ-M1-M2, VDJ-M2, VDJ-CH1-M1-M2) all showed levels of somatic hypermutation similar to membrane bound IGHM and IGHD (Fig. 2C) and far lower than secreted IGHM, IGHA, and IGHG. This suggests that the novel isoforms are likely expressed by naive B cells. Interestingly, alternative splicing was not unique to IGHM. By amplifying bulk B cell cDNA with primers specific to the other isotypes, gel separating distinct bands and Sanger sequencing of said bands, we also identified novel isoforms for IGHA, IGHG, and IGHD, although at lower levels than IGHM (S3 Fig.). Likely due to their low abundance none of these isoforms was detected in single molecule long read sequencing and could therefore not be independently validated.

## Discussion

The highly diverse and repetitive nature of the IGH locus and its transcript has made it hard to analyze. Here we employed several strategies to overcome these difficulties. First, instead of analyzing thousands to millions B cells, we used RNAseq to analyze complete IGH transcripts expressed by single B cells. To accomplish this we utilized the C1 Autoprep System which enables the automated amplification of full length cDNA from single cells which is then fragmented and sequenced on an Illumina sequencer. After sequencing, short reads can be computationally assembled into complete transcripts. Second, in addition to short read RNAseq, we sequenced full length IGH cDNA using the single molecule long read PacBio RS II sequencer. The resulting long reads represent complete IGH transcripts. By combining these technologies we were able to discover and validate a previously unknown splicing event in the IGH transcripts of IGHA isotypes. Instead of one continuous membrane exon (IGHA M), IGHA1 and IGHA2 feature two exons specific to their membrane bound isoform (IGHA M1 and IGHA M2) (S4 Fig.). This brings IGHA transcripts in line with transcripts of all other isotypes that all feature two exons specific to their membrane bound isoform. The IGHA M2 exons are located in the 3’UTR of their respective transcripts and therefore non-coding. The IGHA1/2 3′UTR introns possibly affect the level of membrane bound IGHA protein and are therefore excised from the final membrane IGHA mRNA. Surprisingly, the single molecule read and validation by Sanger sequencing revealed several previously unknown IGH isoforms. The most abundant previously unknown isoforms were of the IGHM isotype and contained membrane exons spliced directly to the VDJ exon. The putative proteins created by these isoforms would not be able to bind an antibody light chain or dimerize with a second heavy chain, yet would possibly still be bound to the B cell membrane (S4 Fig.). These IGHM isoforms have likely escaped discovery until this point in time because commercial and widely used antibodies are raised against the CH1-CH2-CH3 and S exons of secreted IGHM proteins. Therefore the shorter isoforms we detected, which lack these exons in their putative proteins, would be invisible in assays employing these antibodies. Detection will further be complicated by the fact that membrane bound antibody is expressed at levels orders of magnitude lower than secreted antibodies. Future studies aiming to detect the protein products of these novel short isoforms will therefore require highly sensitive assays and custom antibodies raised against the transmembrane region of IGHM.

## Materials and Methods

All study protocols and consent procedures were approved by the Institutional Review Boards at Stanford University. Written consent was obtained from every participant in the study. Blood samples were collected by the Stanford School of Medicine Blood Center. B cell were extracted from Whole Blood using Ficoll (GE Healthcare) gradients combined with Rosette-Sep B-cell (STEMCELL Technologies) separation kits.

### Single Molecule sequencing

Total RNA was extracted from B cells using AllPrep DNA/RNA Mini kits (Qiagen) and cDNA was generated from B-Cell total RNA using SuperscriptIII with oligodT primers containing a tail with a universal priming site. We amplified the cDNA using Hifi Platinum Taq (Life Tech) with a primer for the universal priming site and a primer pool specific for the Leader region of the V segments. Library preparation and sequencing were done following standard Pacific Biosciences (PacBio) protocols. PacBio raw reads processed into circular consensus (CCS) reads using the PacBio pipeline. CCS reads were aligned to the IGH locus using IgBLAST (v.1.2.0)[22] to identify reads containing VDJ recombinations and BLAT (v.35)[23] to align the non VDJ part of the reads to the IGH Locus (NG_001019). 1497 CCS reads contained VDJ recombinations of which we further analyzed 1077 that also contained either complete S or M exons to minimize effects of the mis-priming by the oligodT primer. Data was visualized using custom scripts available upon request.

### Single Cell RNAseq

B cell suspension was loaded onto the Small RNAseq chip for the C1 system (Fluidigm) and RNAseq libraries were prepared following the standard protocol using Clontech and Nextera Reagents (Illumina). Libraries were sequenced on the HiSeq2000 (Illumina). The resulting reads (∼5 million per cell) were trimmed for high quality bases. Trimmed Reads were aligned to the genome using Tophat2[24]. Reads mapping to the IGH Locus were assembled into contigs using SPADES[25]

### Sanger Sequencing

Isoforms where amplified from RNA using Superscript III and Hifi Platinum Taq (Life Tech) using primers targeting the respective exons. Bands were extracted from 2% agarose EX eGels (Life Tech) and if necessary reamplified. Sanger sequencing was performed by Sequetech (Mountain View).

### Primers

To generate amplicon for PacBio sequencing RNA was converted to cDNA using primer Oligo_dT_Universal TGACTGGAGTTCAGACGTGTGCTCTTCCGATCTTTTTTTTTTTTTTTTTTTTTTTTV. We amplified cDNA using Universal_Primer TGACTGGAGTTCAGACGTGTGCTCTTCCGATC and V Leader primer pool (IGHV1/7 ATGGACTGGACCTGGAGGDTC,IGHV1_2 ATGGCTGGAYTTGGAGGATC, IGHV2_1 ATGGACACACTTTGTTCCACGC, IGHV2_2 ATGGACACYTTTGCTMCACACT, IGHV3_1 ATGGAGTTKGGGCTGAGCTG, IGHV3_3 ATGSATTTGKSCTGAGCTGG, IGHV3_4 ATGACGGAGTTTGGGCTGAG, IGHV3_5 ATGGAACTGGGGCTCCGC, IGHV4 ATGAAACACCTGTGGTTCTTCCTC, IGHV5_1 ATGCAAGTGGGGGCCTCTC, IGHV5_2 ATGGGGTCAACCGCCATCC, and IGHV6 ATGTCTGTCTCCTTCCTCATCT). For Sanger sequencing validation of IGHA isoforms primers Val_IGA_CH3_F GACGCTGACGTGCCTGGCAC and Val_IGA_M2_R TGGAAGGCATGTGTAGTAAGGGCG were used. For Sanger sequencing validation of IGHM isoforms primers Val_J4_F ACTGGGGCCAAGGGACCCTGGT and Val_IGM_M2_R GAAGCCTGTGTGAGAGCACACAACT were used. For the identification and validation of low abundance isoforms of IGHD, IGHA and IGHG primers Val_J4_F and Val_IGD_M1_R TGATGAAAGTGACAATGCCGCTGTAG, Val_IGG_M1_R GGAGAAGATCCACTTCACCTTGAAGAAGG, and Val_IGA_M1_R CAGGCTCAGCAGGAAGAGGGTG were used.

## Supporting Information

S1 Fig Validation of IGHA1 and IGHA2 splice variants. a) In an alignment both PacBio read consensus generated from bulk B cell IGH RNA, and single B cell RNAseq read assembly generated of a single IGHA expressing B cells agreed to with the genomic reference in the IGHA1 M1/M2 region. Splice Donor and Splice Acceptor were predicted using SplicePort and agreed with the splice sites determined by PacBio reads and single B cell RNAseq read assembly b) Isoforms were amplified from bulk B cell RNA using primer for exons J4 and IGHA1 M2, gel purified and Sanger sequenced. Sanger sequence and consensus sequence of PacBio reads were aligned to a spliced genomic reference. c) PacBio single molecule sequencing reads mapped to the IGHA2 locus. Reads containing the whole VDJ region as well as either S or M exons were grouped and quantified. As for IGHA1, reads mapping to IGHA2 also showed a splicing separating 2 membrane exons (named IGHA2 M1 and M2)
(PDF)

S2 Fig Validation of IGHM splice variants. Isoform transcripts were amplified from bulk B cell RNA using primer for exons J4 and IGHM M2, gel purified and Sanger sequenced. Sanger sequence and consensus sequence of PacBio reads (if isoforms are represented by more than 2 PacBio reads) were aligned to a spliced genomic reference of isoforms VDJ-CH1-M1-M2, VDJ-M1-M2, and VDJ-M2. Sanger and PacBio consensus reads agreed on splice junction in all cases.
(PDF)

S3 Fig Low abundance IGH splice variants. Isoform transcripts were amplified from bulk B cell RNA using primer for exons J4 and M1 of the respective isotype. Several new isoforms were detected gel purified and Sanger sequenced. Gel images are shown on the left. Schematic representation of isoform splice structure as validated by Sanger sequencing are shown in the middle. Sanger sequences aligned to a spliced genomic reference of isoforms is shown on the right.
(PDF)

S4 Fig Novel exon structure and alternative splicing of the IGH locus. a) PacBio reads of IGHA transcripts and RNAseq read assemblies of single IGHA expressing B cells show that the IGHA1/2 3′UTRs are composed of 2 exons instead of one continuous 3′UTR. b) PacBio reads of IGHM transcripts and Sanger sequencing show several alternative splice variants of the IGHM constant region. If translated, these novel isoforms could serve a unique function on the membrane of likely naive B cells.
(PDF)
