# Supplementary figures and images for: Novel Exons and Splice Variants in the Human Antibody Heavy Chain Identified by Single Cell and Single Molecule Sequencing
Source: PLoS One. 2015 Jan 22;10(1):e0117050. doi: 10.1371/journal.pone.0117050 (PMC4303433; doi:10.1371/journal.pone.0117050)

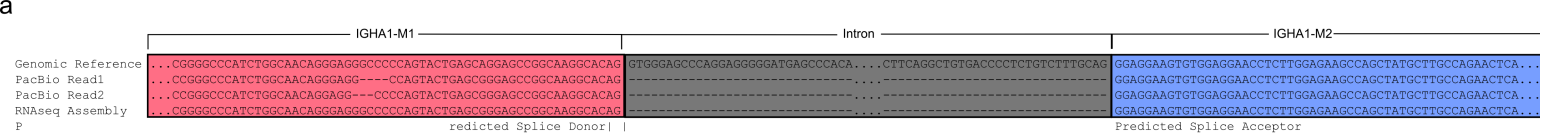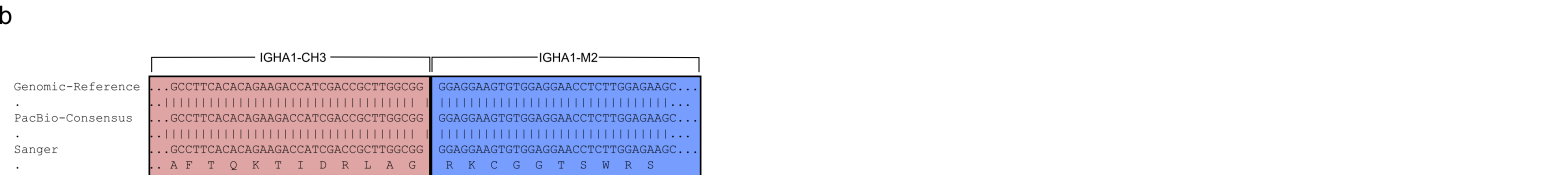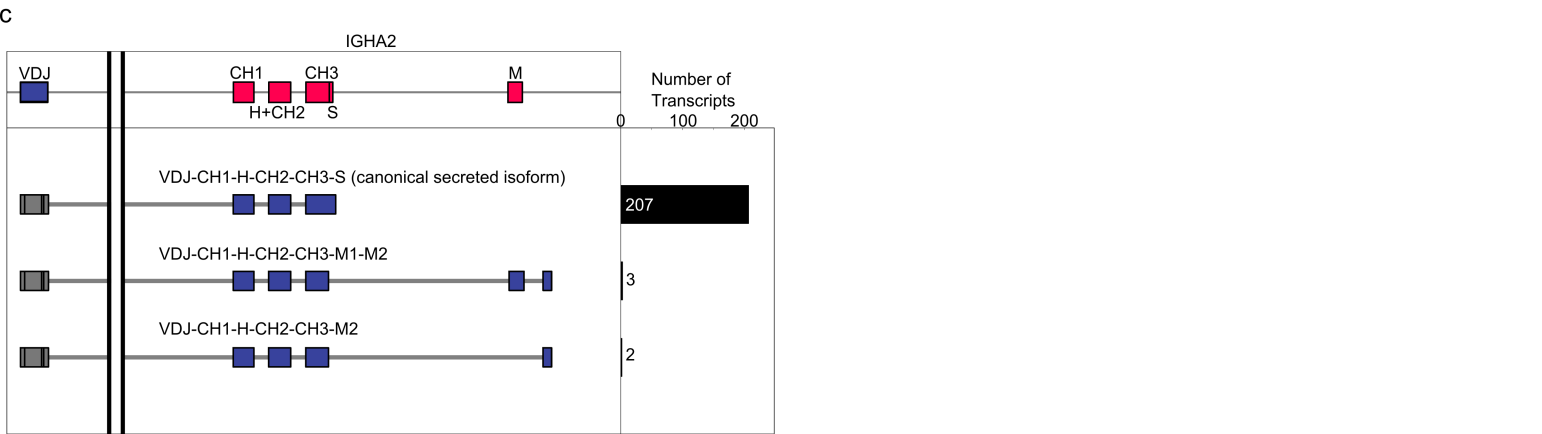

Supplement: S1 Fig — a) In an alignment both PacBio read consensus generated from bulk B cell IGH RNA, and single B cell RNAseq read assembly generated of a single IGHA expressing B cells agreed to with the genomic reference in the IGHA1 M1/M2 region. Splice Donor and Splice Acceptor were predicted using SplicePort and agreed with the splice sites determined by PacBio reads and single B cell RNAseq read assembly b) Isoforms were amplified from bulk B cell RNA using primer for exons J4 and IGHA1 M2, gel purified and Sanger sequenced. Sanger sequence and consensus sequence of PacBio reads were aligned to a spliced genomic reference. c) PacBio single molecule sequencing reads mapped to the IGHA2 locus. Reads containing the whole VDJ region as well as either S or M exons were grouped and quantified. As for IGHA1, reads mapping to IGHA2 also showed a splicing separating 2 membrane exons (named IGHA2 M1 and M2) (PDF) [file pone.0117050.s001.pdf]

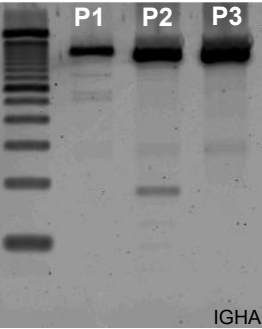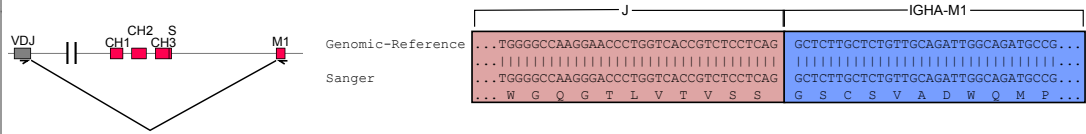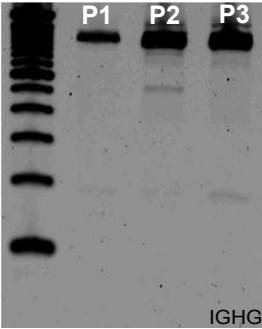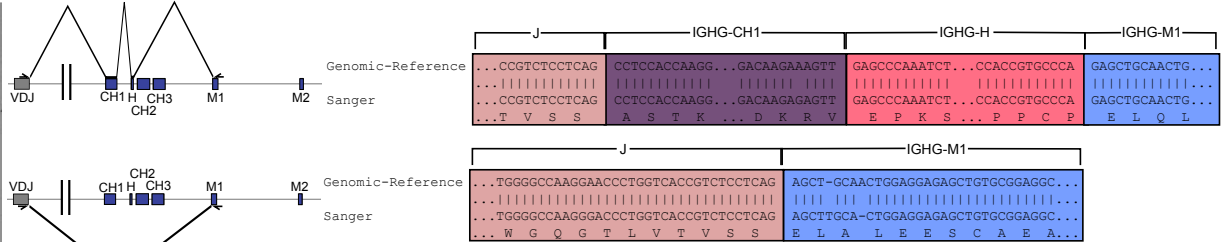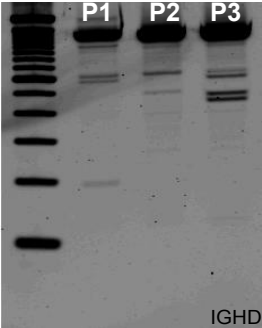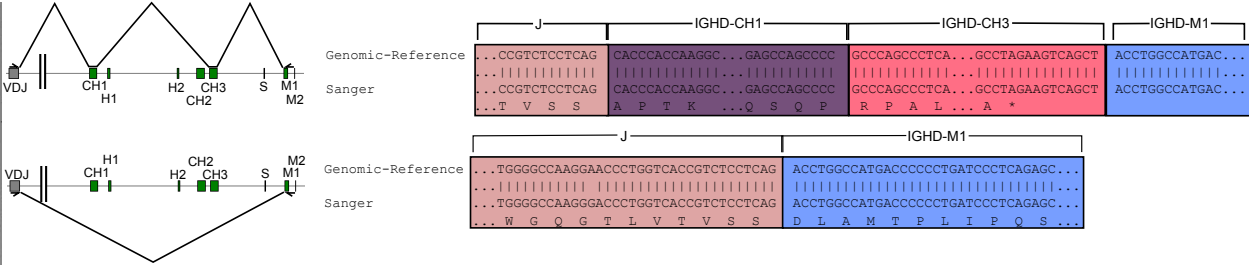

Supplement: S3 Fig — Isoform transcripts were amplified from bulk B cell RNA using primer for exons J4 and M1 of the respective isotype. Several new isoforms were detected gel purified and Sanger sequenced. Gel images are shown on the left. Schematic representation of isoform splice structure as validated by Sanger sequencing are shown in the middle. Sanger sequences aligned to a spliced genomic reference of isoforms is shown on the right. (PDF) [file pone.0117050.s003.pdf]

**a**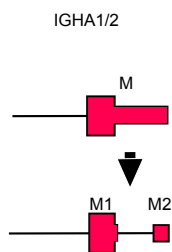**b**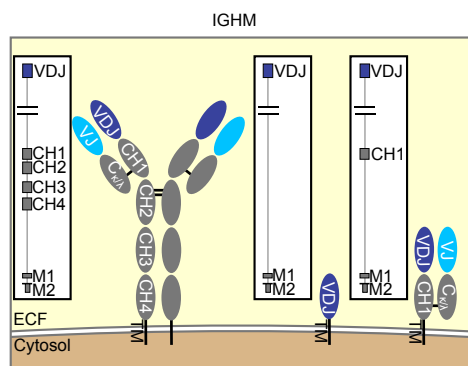

Supplement: S4 Fig — a) PacBio reads of IGHA transcripts and RNAseq read assemblies of single IGHA expressing B cells show that the IGHA1/2 3′UTRs are composed of 2 exons instead of one continuous 3′UTR. b) PacBio reads of IGHM transcripts and Sanger sequencing show several alternative splice variants of the IGHM constant region. If translated, these novel isoforms could serve a unique function on the membrane of likely naive B cells. (PDF) [file pone.0117050.s004.pdf]
