# Supplementary material for: Novel Exons and Splice Variants in the Human Antibody Heavy Chain Identified by Single Cell and Single Molecule Sequencing
Source: PLoS One. 2015 Jan 22;10(1):e0117050. doi: 10.1371/journal.pone.0117050 (PMC4303433; doi:10.1371/journal.pone.0117050)
Supplement: S2 Fig — Isoform transcripts were amplified from bulk B cell RNA using primer for exons J4 and IGHM M2, gel purified and Sanger sequenced. Sanger sequence and consensus sequence of PacBio reads (if isoforms are represented by more than 2 PacBio reads) were aligned to a spliced genomic reference of isoforms VDJ-CH1-M1-M2, VDJ-M1-M2, and VDJ-M2. Sanger and PacBio consensus reads agreed on splice junction in all cases. (PDF) [file pone.0117050.s002.pdf]

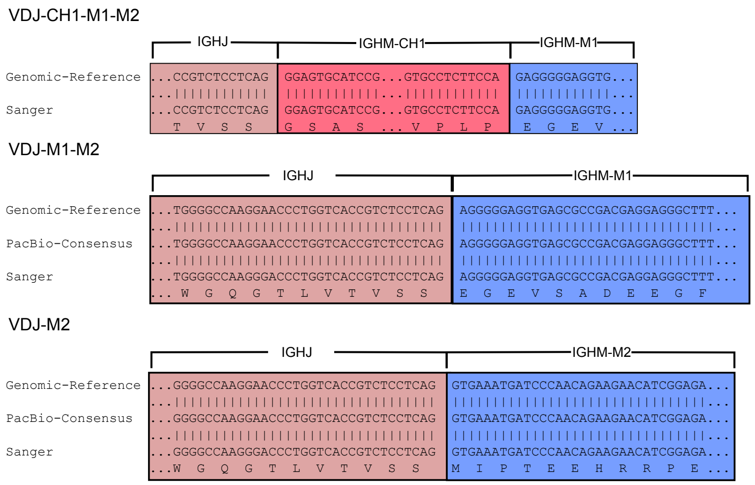

VDJ-CH1-M1-M2
IGHJ
IGHM-CH1
IGHM-M1
Genomic-Reference ...CCGTCTCCTCAG GGAGTGCATCCG...GTGCCTCTTCCA GAGGGGGAGGTG...
Sanger ...CCGTCTCCTCAG GGAGTGCATCCG...GTGCCTCTTCCA GAGGGGGAGGTG...
T V S S G S A S ... V P L P E G E V ...
VDJ-M1-M2
IGHJ
IGHM-M1
Genomic-Reference ...TGGGGCCAAGGAACCCTGGTCACCGTCTCCTCAG AGGGGGAGGTGAGCGCCGACGAGGAGGGCTTT...
PacBio-Consensus ...TGGGGCCAAGGAACCCTGGTCACCGTCTCCTCAG AGGGGGAGGTGAGCGCCGACGAGGAGGGCTTT...
Sanger ...TGGGGCCAAGGGACCCTGGTCACCGTCTCCTCAG AGGGGGAGGTGAGCGCCGACGAGGAGGGCTTT...
... W G Q G T L V T V S S E G E V S A D E E G F
VDJ-M2
IGHJ
IGHM-M2
Genomic-Reference ...GGGGCCAAGGAACCCTGGTCACCGTCTCCTCAG GTGAAATGATCCCAACAGAAGAACATCGGAGA...
PacBio-Consensus ...GGGGCCAAGGAACCCTGGTCACCGTCTCCTCAG GTGAAATGATCCCAACAGAAGAACATCGGAGA...
Sanger ...GGGGCCAAGGGACCCTGGTCACCGTCTCCTCAG GTGAAATGATCCCAACAGAAGAACATCGGAGA...
...W G Q G T L V T V S S M I P T E E H R R P E ...
